# Supplementary material for: Using social choice theory and acceptability analysis to measure the value of health systems
Source: PLoS One. 2020 Jul 2;15(7):e0235531. doi: 10.1371/journal.pone.0235531 (PMC7332049; doi:10.1371/journal.pone.0235531)
Supplement: Attachment — Submitted filename: Response to reviewer 2.docx [file pone.0235531.s001.docx]

Many thanks for your valuable comments. We have accordingly made revisions and marked them using RED.

Comments to the Author

1. Is the manuscript technically sound, and do the data support the conclusions?

The manuscript must describe a technically sound piece of scientific research with data that supports the conclusions. Experiments must have been conducted rigorously, with appropriate controls, replication, and sample sizes. The conclusions must be drawn appropriately based on the data presented.

Reviewer #2: Partly

2. Has the statistical analysis been performed appropriately and rigorously?

Reviewer #2: Yes

3. Have the authors made all data underlying the findings in their manuscript fully available?

The PLOS Data policy requires authors to make all data underlying the findings described in their manuscript fully available without restriction, with rare exception (please refer to the Data Availability Statement in the manuscript PDF file). The data should be provided as part of the manuscript or its supporting information, or deposited to a public repository. For example, in addition to summary statistics, the data points behind means, medians and variance measures should be available. If there are restrictions on publicly sharing data—e.g. participant privacy or use of data from a third party—those must be specified.

Reviewer #2: Yes

4. Is the manuscript presented in an intelligible fashion and written in standard English?

PLOS ONE does not copyedit accepted manuscripts, so the language in submitted articles must be clear, correct, and unambiguous. Any typographical or grammatical errors should be corrected at revision, so please note any specific errors here.

Reviewer #2: Yes

5. Review Comments to the Author

Please use the space provided to explain your answers to the questions above. You may also include additional comments for the author, including concerns about dual publication, research ethics, or publication ethics. (Please upload your review as an attachment if it exceeds 20,000 characters)

Reviewer #2: Positive comments:

The paper covers an interesting topic with improving a method to appropriately address the several factors that are relevant for the value of different health systems. Following an approach that takes rankings of these factors into account instead of weighting them equally seems a reasonable approach.

The overall logic of the paper is understandable. The problem with the existing methodology (computing the average overall) and the aimed contribution is pointed out. In general, the development of the methodology in the empirical part (section 2) is reasonable. Moreover, it is clearly a plus, that the author includes detailed information about the used software (e.g., for the SMAA-2) and the tested data set. This makes the method also applicable for researchers and practitioners in other contexts for similar problems.

The general demands from the journal are met, in that the study presents the results of original research, the methodology is overall described in a reasonable manner, conclusions seem to be supported by the data (at least partly, although there are some issue with this method outlined below) and standards of data and methodological transparency are obtained.

However, there is some potential to improve the paper, as pointed out below.

Methodological Development (Section 2)

The method of using ranked weights to improve the value measurement seems to be solid and bases on an established approach that is motivated from former research. However, some open questions and issues remain.

First, it is not clear, whether or how it is accounted for the several sub-metrics that build the metrics Satisfaction, Access and Efficiency. Are they still aggregated using the average method or are ranked weights used for them as well? If they are still aggregated, there would still be the issue of information loss within these metrics. Is there an approach or argument to deal with this issue?

Many thanks for your valuable comments. The scores for each sub-metric are arithmetically averaged to calculate each metric sore and those scores are then arithmetically averaged to construct the Value Measure. The proposed method is reasonably applicable to both aggregation scenarios. Because of the data availability, this paper only re-builds the Value Measure using the scores of Satisfaction, Access and Efficiency.

Second, it remains unclear, how feasible this methodology is for a large set of indicators with many possible preference rankings. It appears to require a high amount of computational power and might get complex if many indicators are present. This limitation raises concerns about its applicability for a broader range of (more complex) problems. It would be helpful to add a statement of anticipated generalizability of the method.

Many thanks for your valuable comments. The application of our method could be more complicated when there exist more metrics to consider, since the importance orders would dramatically increase as the increase of the number of metrics. Future research should develop some statistical techniques, for example, principal component analysis, to select useful orders. This statement is added in the Conclusion section to discuss future research directions.

Moreover, the argumentation and description of the methodological development (section 2) should be improved. Some decisions in the development are (apparently) made arbitrarily in that they are not justified convincingly. For the following points, the author should outline their reasons leading to these methodological decisions more clear:

1. Why is it necessary to normalize values, since the original metrics in the FHI are already normalized (on a scale from 0-100)?

Many thanks for your valuable comments. Normalization is a fundamental work to implement various multiple criteria decision making techniques, the primary purpose of which is to alleviate the adverse impact of data magnitude.

2. Why is a uniform and a Gaussian distribution used?

Many thanks for your valuable comments. Following the work of Yang et al. (2012) (F Yang, S Ang, Q Xia, C Yang, Ranking DMUs by using interval DEA cross efficiency matrix with acceptability analysis, European Journal of Operational Research, 223(2), 483-488), we investigate both Uniform and Gaussian distribution. In addition, we propose to consider other statistical distributions in future research.

3. The introduction of surrogate weights is very short and the mathematical calculation of them appears 2 pages after their introduction. This disrupts comprehension. It could also contribute the quick comprehension of the methodology, if the author could add a short explanation when introducing some new parameters (e.g., what k stands for (p. 7)).

Many thanks for your valuable comments. This section aims to determine the closed form solutions to the pessimistic and optimistic results for DMU $i$ under certain individual preference. We introduce the auxiliary variable $k$ to simplify the derivation.

Results of Empirical Analysis (Section 3)

The final comparison between the developed and the previous method to evaluate the value of health systems bases on Spearman’s Rank correlations. However, there are several issues with the presentation of this analysis:

1. It is not designated if the Spearman rank correlations are statistically significant.

2. The author provides a proportional improvement of the summed correlation coefficients, but does not provide a statistical index of whether this improvement (in %) is a significant improvement. The argumentation would be more convincing if an objective index would be added that proves a significant improvement in comparison to the previous method of averaging over metrics.

3. It is questionable whether the correlation with ranks based on the averaged metrics is appropriate, since it is argued in the paper before that averaging over indices has some methodological issues. Taking the ranks developed by the averaged metrics then as a comparison standard seems inconsistent with that former argumentation.

As such, it remains unclear, how to evaluate the improvement of the revised value measure in comparison to the former approach. For these reasons the soundness of conclusions based on data can be only confirmed partly.

Many thanks for your valuable comments. In statistics, Spearman’s rank correlation coefficient is a nonparametric measure between the rankings of two variables, and evaluates how well the relationship between two variables can be described using a monotonic function. The Spearman's rank correlation coefficient is capable of reflecting the conflict between ranking orders (Zhou et al. 2006). The more discordant the rankings of two variables, the smaller the Spearman’s rank correlation coefficient (Diakoulaki et al. 1995). Motivated by Lyerly (1952), we calculate and compare the average Spearman’s rank correlation coefficients to measure the strength and direction of association between obtained ranks and variables, and assessing the accuracy of models. In this sense, Spearman’s rank correlation coefficients have been extensively used to verify the advantage of proposed method (Wu et al., 2018). In addition, to the best of our knowledge, the significance level is usually obtained from computing the Pearson Correlation Coefficient, not for Spearman’s rank correlation coefficient.

Tables and Figures

The Tables and Figures are helpful and improve understanding in general.

For tables, however, information about the depicted measure could help to intuitive understanding them. Although mentioned in the text, this information should be included directly in the table or the title of the table or added as a footnote (see for example Table 4, 5, 7). In Table 7 significance of correlation coefficients should be added (see also comment above).

It would be nice, if information from Figure 1 and 2 could be joined allowing a direct comparison between both distributions.

Many thanks for your valuable comments. We compare the results obtained from both distributions in Table 6. It is somehow confusing when combine Figures 1 and 2.

Language

The author could improve wording and language use at some points to make her points really clear:

For example the sentence “Such an investigation sheds much-needed light on potential incentives and directions for academic, managerial and policy-related implications.” (p. 5) does not point out which implications are derived. The sentence “For the purpose of measuring the performance previously aggregated using the arithmetic average” (p. 5) is misleading, as it suggests that the former performance of the value measure is investigated, which is not the aim nor the outcome of the paper (instead, it compares the correlations of the sub-metrics with the old and the new values measure.

Furthermore, when describing the metrics of the FHI, the terms “sub-metric” and “metric” (introduced on p. 3) are used, but later on in the methodological part the term “sub-indices” refers to the metrics Access, Satisfaction and Efficiency, which can be confusing. It should be stated clearly once, which concepts from the application case in the empirical part refer to which concepts in the developed methodology.

Another issue is that some sentences in the introduction are nearly adopted literally, but are not marked as verbatim quotes. Concretely these are the following sentences:

“The challenges of delivering health care in many countries are receiving increasing attentions as costs continue to rise and evidence of uneven quality accumulates” (p. 2, first sentence, adopted from Porter, 2008), and “The Future Health Index (FHI)1 is a research-based platform designed by the Royal Philips to help determine the readiness of countries to address global health challenges and build sustainable...” (adopted from the FHI website). Both should be marked as verbatim quotes.

There is an (important) typo in Table 1 (p. 4). It is “healthy life expectancy” instead of “health life expectancy”.

Many thanks for your valuable comments. We have made revisions according to your comments.

Structure

In general the structure and the development of the paper makes sense. However, maybe the author could avoid repeating information and foster a fluent reading when slightly restructuring the paper.

In the current draft, FHI is described in detail already in the introduction, then the general methodological approach is developed and then the empirical section is introduced again with a description of the FHI. The detailed description of the FHI could also be positioned as an own section before the empirical section or included in the empirical section.

Many thanks for your valuable comments. We present the background of FHI in the Introduction section. The empirical study uses the data of FHI for demonstration. Based on your comments, we divide the empirical study section into two sub-sections: FHI 2018 and Result and Analysis.

In the introduction, the objective and contribution of the study is mentioned relatively late (after introduction of the FHI). This lets the reader in the dark about the purpose of the study and disrupts a proper understanding from the very beginning.

Many thanks for your valuable comments. The logic of our introduction is firstly describing the concept and development of FHI; then discussing the shortcomings of Value Measure; lastly presenting the objective and contribution of our study. We believe that this organization makes the reading and understanding easy.

Outline of Contribution

Although the paper apparently focuses on the development of a methodological approach, the motivation of the paper addresses several content-wise issues that are not really addressed later on in the paper. The author should become more clear what exactly the problem is they aim to address and to solve with their research (otherwise it appears like overstating the contribution of the paper).

As such, it remains unclear how applicable the findings are and which exact practical implications can be derived from them. It would contribute to the value of the paper from a broader perspective if the author would answer the following questions when shortly discussing the results:

1. How do the findings and the improvement of measurement contribute to achieving high value for various stakeholders?

2. Which (if any) content-wise and/or practical implications can be derived from the improved value measurement?

Many thanks for your valuable comments. We address these two issues in the Result and Analysis section.

Concluding Remarks

Taking together, the paper covers an important topic with an immense societal relevance. It uses a convincing methodological approach that is theoretically reasoned to improve the value of health systems.

Major issues in the current draft and important implications for the further development of the paper will be to improve the scientific proof of empirically testing the improvement of the revised measure of the FHI in comparison to the former approach of averaging the conducted metrics. Moreover, the practical implications and methodological applicability of the results should be worked out more clearly.

Some concerns are also raised through the use of nearly verbatim quotes from other sources without designating them as such.

I wish the author good luck and look forward to the further development of the paper.

6. PLOS authors have the option to publish the peer review history of their article (what does this mean?). If published, this will include your full peer review and any attached files.

If you choose “no”, your identity will remain anonymous but your review may still be made public.

Do you want your identity to be public for this peer review? For information about this choice, including consent withdrawal, please see our Privacy Policy.

Reviewer #2: No
