# Supplementary material for: Using social choice theory and acceptability analysis to measure the value of health systems
Source: PLoS One. 2020 Jul 2;15(7):e0235531. doi: 10.1371/journal.pone.0235531 (PMC7332049; doi:10.1371/journal.pone.0235531)
Supplement: Attachment — Submitted filename: Response to reviewer 3.docx [file pone.0235531.s002.docx]

Many thanks for your valuable comments on our manuscript. We make the revisions and highlight them in the revised version using BLUE.

Reviewer #3: This communication presents an interesting novel method for measuring the value of health systems.

As per reviewers' suggestions, the authors have revised the paper successfully, but some grammatical errors are still present in the revised manuscript. Improve the errors.
